# Potent Stimulation of Blood Flow in Fingers of Volunteers after Local Short-Term Treatment with Low-Frequency Magnetic Fields from a Novel Device

**DOI:** 10.1155/2014/543564

**Published:** 2014-05-21

**Authors:** Richard H. W. Funk, Lilla Knels, Antje Augstein, Rainer Marquetant, Hermann F. Dertinger

**Affiliations:** ^1^Institute for Anatomy, Medical Faculty, Dresden University of Technology, Fiedlerstraße 42, 01307 Dresden, Germany; ^2^Medical Clinic/Cardiology, Department of Molecular Cardiology, Medical Faculty, Dresden University of Technology, Fiedlerstraße 42, 01307 Dresden, Germany; ^3^Institute for Biological Interfaces, Research Centre Karlsruhe, Kaiserstraße 12, 76131 Karlsruhe, Germany

## Abstract

A novel hand-held low-frequency magnetic stimulator (MagCell-SR) was tested for its ability to stimulate microcirculation in fingers of healthy volunteers. Blood flow during and after 5 minutes exposure was quantified using Laser Doppler Perfusion Imaging Technique. The device was positioned between the wrist and the dorsal part of the backhand. Because the increase in blood flow could be caused by a release of nitric oxide (NO) from the vascular endothelial cells we tested NO production with a fluorescence marker and quantified the measurements in cell cultures of human umbilical endothelial cells (HUVEC). Exposure increased blood flow significantly, persisted several minutes, and then disappeared gradually. In order to assess the effect of a static magnetic field, the measurements were also carried out with the device shutoff. Here, only a small increase in blood flow was noted. The application of the rotating MagCell-SR to the HUVEC cultures leads to a rapid onset and a significant increase of NO release after 15 minutes. Thus, frequencies between 4 and 12 Hz supplied by the device improve microcirculation significantly. Therefore, this device can be used in all clinical situations where an improvement of the microcirculation is useful like in chronic wound healing deficits.

## 1. Introduction

A critical review of electromagnetic therapy by Glaser [1, 2] arrives at the conclusion that most effects seen, in particular with magnetic fields, do not stand up to rigorous scientific examination. This is also true for many of the pulsating electromagnetic field types (PEMF), which have been extensively studied. Here, the main point of criticism is that the magnetic fields applied would generally be too weak to induce electric fields of therapeutic relevance.

This raises two questions: (i) is the induced electric field, in fact, the major driving force for biomedical effects and (ii) does a lower threshold of field strength exist, which has to be overcome in order to provoke a statistically significant biological effect?

The answer for both questions is yes. Using 50 Hz magnetic and electric fields, Schimmelpfeng and Dertinger [3] found identical stimulation of the cellular second messenger cyclic AMP for both field types. Concerning a possible threshold, it can be inferred from published data that the flux density of the magnetic field should be at least 2 mT, corresponding to an induced field strength of 4 to 8 mV/m, in order to get a significant biological response [4, 5].

These considerations led to the development of a powerful magnetic stimulator for local application, delivering suprathreshold flux densities even at a distance (tissue depth) of 3 to 4 cm. In the following we present a study with healthy volunteers, showing that this device improves blood flow to a statistically highly significant extent. To investigate further the cause of the putative microvessel dilation we tested the MagCell-SR in HUVEC cultures and could show a significant increase of NO release after application.

## 2. Methods

### 2.1. Healthy Volunteer Experiments

**Figure 1:**
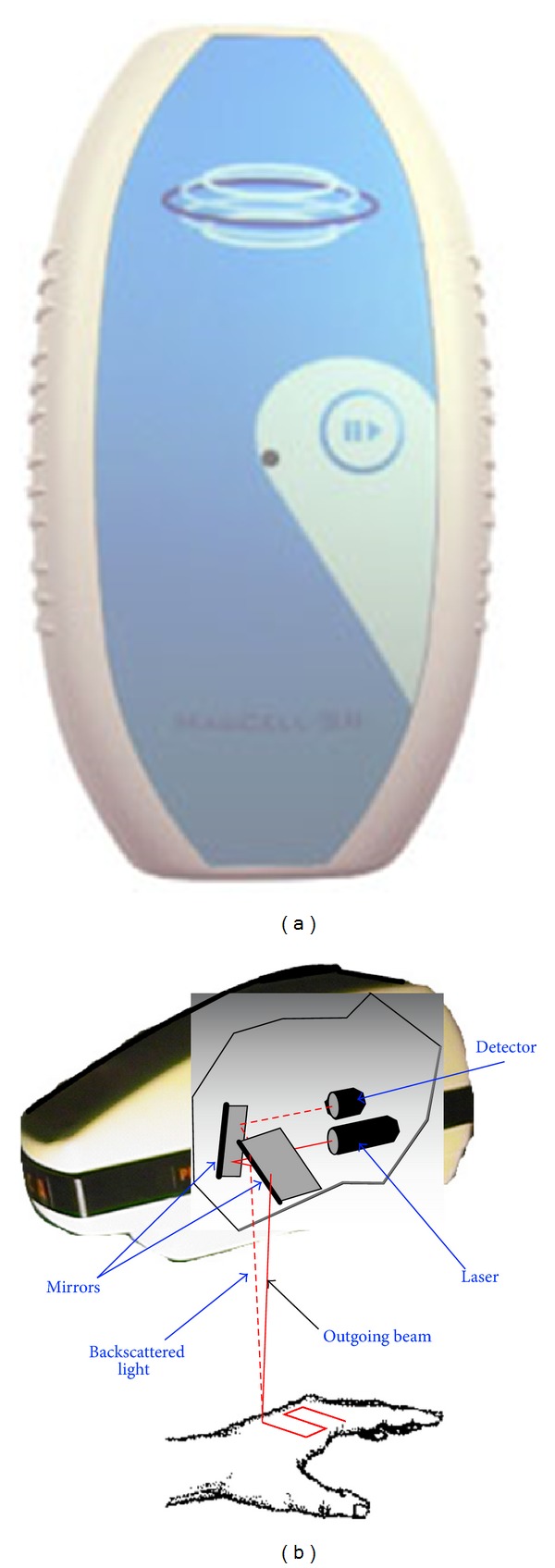
(a) MagCell hand-held therapy device. (b) Principle of PeriScan Laser Doppler Perfusion Imaging (LDPI) System. (Picture is taken from the PeriScan User-Manual, Part 44–00079-07; revised June 2004, SP, Perimed.)

Short-term treatments (5 minutes) were carried out with the MagCell-SR device using rotating strong magnets (Fa. Physiomed, Laipersdorf, Germany) (Figure 1(a)) exhibiting electromagnetic frequencies between 4 and 12 Hz. A comparison of this treatment was also made with static magnetic fields (shut off the device means no rotation of the magnet disc).

Blood flow was recorded during and after exposure using noninvasive Laser Doppler Perfusion Imaging (LDPI) Technology (PeriScan PIM Perfusion Imager, Perimed AB, Stockholm, Sweden) (Figure 1(b)). Different protocols for exposure conditions and data acquisition were used (A–E, see Table 1).

The distance between scanner head and skin ranged from 10 to 12 cm. The sampling depth of the laser beam is typically 300–500 *μ*m in skin tissue. The region of interest (ROI) was a square of 5 cm × 5 cm positioned with the guidance of tape markers.

The volunteers (see Table 1 for details) were adapted to the ambient temperature. For exposure, the MagCell device, covered by a 1 cm thick layer of foamed plastic, was positioned between the wrist and the dorsal part of the backhand. The magnetic flux density at the site of exposure ranged from 10 to 15 mT.

**Figure 2:**
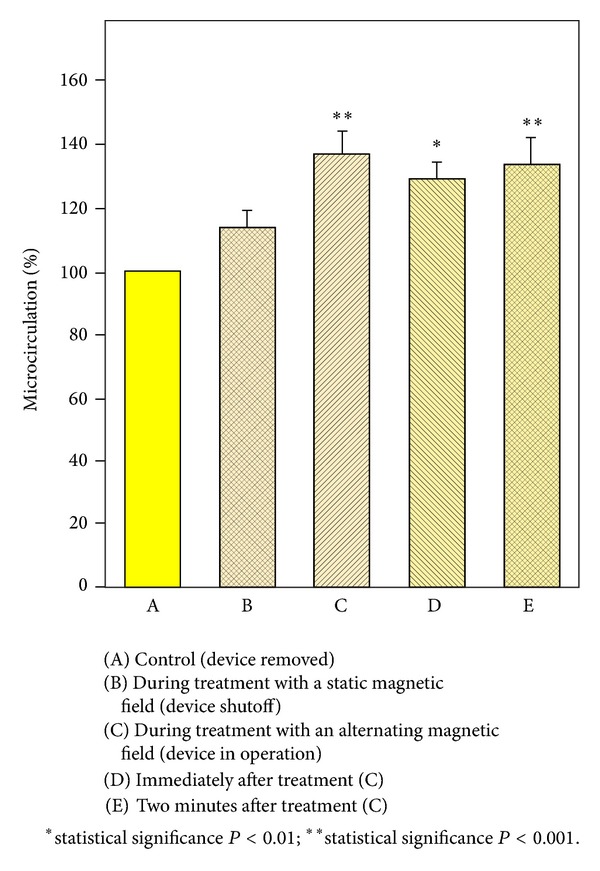


Recorded images were analyzed using image processing software (Perimed). The ROI was expressed in arbitrarily units (see example given in Figure 2). Data from the different protocols were compared using Student-Newman-Keuls Multiple Comparisons Test.

The study protocol with healthy volunteers was approved by the Ethics Committee of the Medical Faculty of the TU Dresden.

### 2.2. *In Vitro* Experiments

Endothelial cells derived from veins of human umbilical cords were cultured as described elsewhere [6].

For* ROS *detection, the cells were viewed with differential interference contrast (DIC) and in fluorescence mode with DAF-2DA (diaminofluorescein diacetate, Molecular Probes) [5 *μ*M in medium) at 37°C with 5% CO_2_ in a dark and humidified box for at last 45 min. Cells were viewed with a IX81 fluorescence microscope (Olympus Biosystems) equipped with an OBS CCD FV2T camera (Olympus Europa GmbH), photo documented, and analyzed using cell^∧^R by Olympus. All experiments were done at least six times.

### 2.3. RNA Isolation and Real-Time PCR

30 minutes after isolation and incubation of RNA from HUVECs, reverse transcription (RT) and real-time PCR were performed as extensively described by Poitz et al. [7] using the following primers for real-time PCR: 5′-GATGGTGACTTTGGCTAGCTGG-3′ eNOS-forward (human nitric oxide synthase 3), 5′-TCCTGGAGGATGTGGCTGTC-3′ eNOS-reverse (human nitric oxide synthase 3),  5′-CATGAGCAAAGGCGCAGAAC-3′ iNOS-forward (human nitric oxide synthase 2), 5′-CCTGGCAATGGAGAGAAACTG-3′ iNOS-reverse (human nitric oxide synthase 2), 5′-TTGCGACCTTGACCATCTTTG-3′ HPRT1-forward (hyperparathyroidism 1), 5′-CTTTGCTGACCTGCTGGATTAC-3′ HPRT1-reverse (hyperparathyroidism 1).

### 2.4. Flow Cytometry

Directly after the 5 minutes MagCell stimulation the cells were incubated with DAF-2-DA at a final concentration of 10 *μ*M for 30 min at 37°C in the dark. Excess dye was removed by washing in PBS. Fluorescence intensity was measured in a FACSCalibur cytofluorometer (Becton-Dickinson, Heidelberg, Germany). For each analysis, 10,000 cells were recorded. Experiments were performed in triplicate.

## 3. Results

### 3.1. Healthy Volunteer Experiments

Table 1 compiles the numerical data for the different exposure protocols and their statistical analysis. Control values were normalized to 100%. Maximum stimulation of blood flow to 137% of the control is achieved during treatment and is of high statistical significance (*P* < 0.001). This also holds true for the data obtained immediately after treatment (*P* < 0.01) and 2 minutes after treatment (*P* < 0.001). The differences between these 3 data groups (C, D, E) are, however, not statistically significant (*P* > 0.05).

Thus, 5 minutes stimulation with device in operation is stable, even for some time after exposure. A slight stimulation (114%) can also be achieved with the static magnetic field (B; device shutoff, magnets do not rotate), but this effect is not statistically significant (*P* > 0.05).

**Figure 3:**
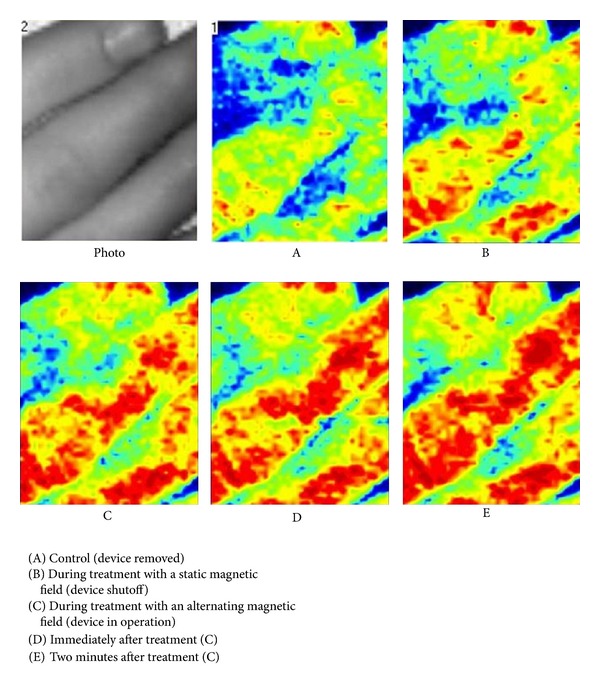
Stimulation of microcirculation by the MagCell-SR device. (Laser Doppler Perfusion Imaging Technique.)

A bar plot of these data is given in Figure 2. Finally, Figure 3 shows a coloured representation of the region of interest.

### 3.2. *In Vitro* Experiments

**Figure 4:**
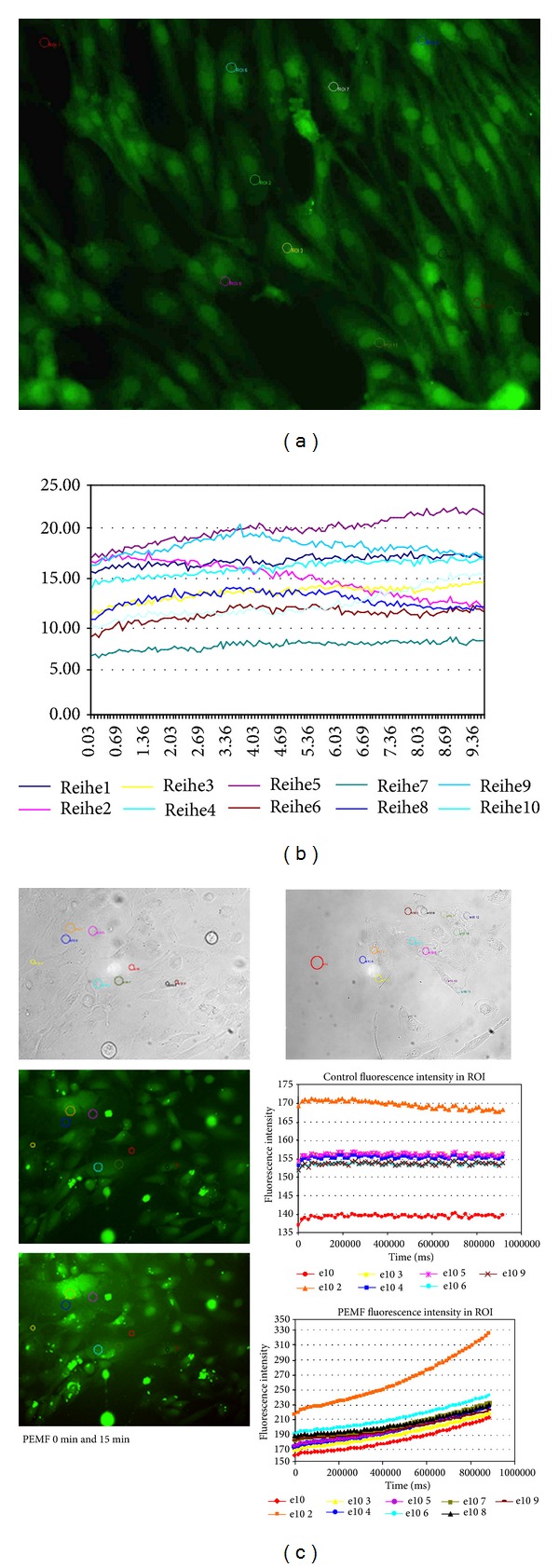
(a) Differential interference contrast (DIC) pictures of HUVEC cells. (b) DAF-2DA fluorescence picture of HUVEC cells (circles indicate the measurement points—regions of interest depicted in the MagCell experiment are depicted in (c)). (c) Example of a MagCell stimulation experiment.

**Figure 5:**
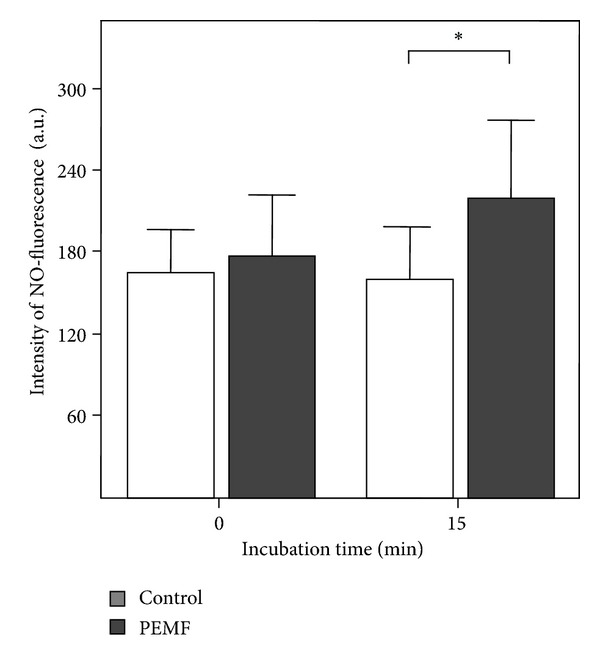
MagCell stimulation experiment (in the left row DIC and fluorescence pictures before and 15 min after stimulation; right row: control DIC and control ROI measurements; below: ROIs of stimulation with PEMF).

Figures 4 and 5 show the DIC and DAF-2DA fluorescence images before and after PEMF stimulation. The coloured circles in the pictures indicate different regions of interest (ROIs) where the quantitative measurements (fluorescence intensity correlating with the amount of released NO) were performed.

A clear increase in NO production can be found in most of the ROIs (Figure 5). Interestingly, a clear stimulation of NO release was only found in subconfluent monolayers or at least groups of spindle shaped HUVEC (Figures 4 and 5) not in separated single cells with a flat and rounded shape (not shown). Only these cultures were taken for quantitative evaluations.

The mean values of our measurements (*n* = 9 cultures in controls and measurements, each with 5–9 ROIs, means 56 points of measurements) show a significant 24% (*P* < 0.001) increase of NO release (Table 2).

**Figure 6:**
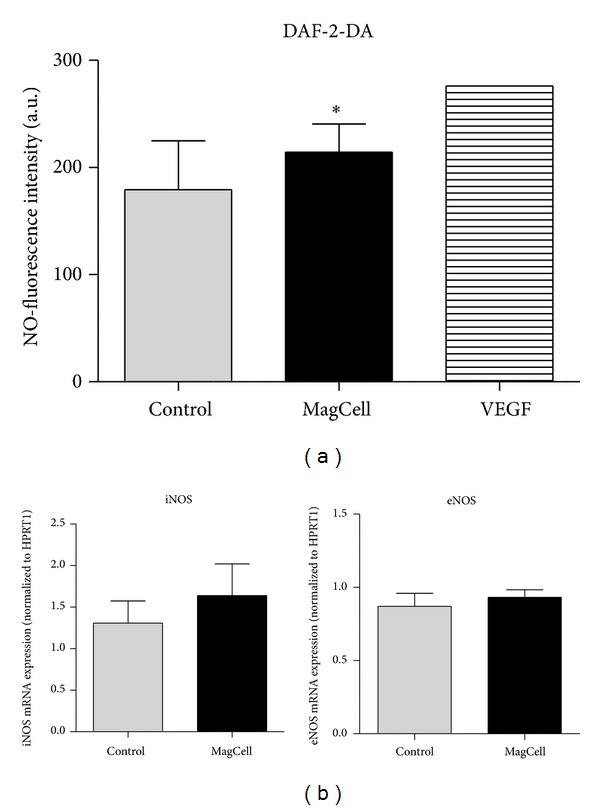
(a) Flow cytometric analysis of human umbilical endothelial cells (HUVECs), 5 min after 5 min MagCell stimulation (*n* = 3). Controls: cells without MagCell stimulation (*n* = 3), or after addition of VEGF (*n* = 1). Asterisk: *P* = 0,120 (ANOVA Test, post hoc Bonferroni). (b) Detection of iNOS and eNOS expression by real-time PCR in HUVECs 30 min after 5 min MagCell stimulation or in unstimulated HUVECs (control); *n* = 3.

Flow cytometric studies were made indicating the DAF-2DA fluorescence intensity correlation with the amount of released NO. Here we could also detect an increase in fluorescence with a moderate significance (Figure 6(a)).

RT-PCR measurements revealed that on the level of mRNA expression a moderate but not significant increase could be found for the endothelial NO-synthetase (eNOS) as well as for the inducible NO-synthetase (iNOS) (Figure 6(b)).

## 4. Discussion

Having a rotating disc with strong magnets, this small device is able to apply electromagnetic fields strong enough to induce significant tissue reactions. Therefore, this construction is superior to other devices which are either too weak to reach measurable field strengths or use direct electric stimulation: no electrodes have to be used, thus avoiding electric sensations or skin irritations. It is small and does not need large coils or power supplies. Moreover, it acts through clothes and bandages. This is an important feature, when the device is applied, for example, for wound healing, which, according to the results discussed here, can be expected to yield good results. Finally, the strong magnetic field provided by the device enables stimulation of deep seated tissue and can even penetrate into bone. Thus, this device should also be used in all clinical situations where an improvement of the microcirculation is meaningful like, for example, in chronic wound healing deficits and in other chronic and degenerative diseases of the skeleton or in the peripheral nervous system.

The results of this study unequivocally prove, that powerful electromagnetic stimulation of microcirculation is possible with magnetic fields of suprathreshold flux density as provided by the MagCell device and that the effect even persists several minutes after termination of the exposure. That the induced electric field is, as outlined in Section 1, the relevant driving force for microcirculation, is further strengthened by a volunteer study by Wikstr$\ddot{\text{o}}$m et al. [8]. Using Laser Doppler Imaging Technique, these authors investigated the influence of transcutaneous nerve stimulation (TENS) upon microcirculation in the skin, which was found to increase by 40%. This effect is almost identical with the figure of 37% obtained in our study and suggests that the induced electric field (current density) is involved in the effect rather than the magnetic field itself.

Microvessel dilatation is the most likely mechanism to explain our data. This is, for example, supported by the study of Smith et al. [9] on rats. These authors found local PEMF stimulation to increase the diameter of arteriolar microvessels by 9%. According to the law of Hagen-Poiseuille, this amount of vasodilatation can easily explain a flow increase of about 40%.

In addition, other factors like stimulation of the metabolic demand of the cells in the skin, muscle, and bone can indirectly lead to enhanced blood flow, too. It is known that PEMF can increase proliferation rates in cell lines (murine osteosarcoma, [10]), in chondrocytes [11] and in osteoblasts [12, 13]. PEMF is also able to trigger mitosis and differentiation in stem cells (in the adult rat brain; see [14]). Diniz et al. [15] could show that the stimulatory effect of PEMF on osteoblast proliferation and differentiation was mediated by an increase in nitric oxide (NO) synthesis.

NO exerts many important functions on the vascular wall in addition to its vasodilatory effect. These includes suppressing the inflammatory response induced by cytokines, Spiecker et al. [16]; inhibiting apoptosis, Dimmeler and Zeiher [17]; and regulating cell migration and angiogenesis, Murohara et al. [18].

The stimulated increase in NO production by the HUVEC cultures in our* in vitro* experiments can explain very well the observed stimulation of peripheral blood flow* in vivo*. Also the rapid onset of the NO release in the first minutes correlates very well with the blood flow increase during and after 2 minutes of application in the* in vivo* experiments. Only in spindle shaped typical HUVEC cells such clear significant NO increase could be found, not in the separated single cells, presumably being less differentiated and being under stress.

A more summary, although statistical with a much higher amount of cells, approach is flow cytometry, which shows also a moderate NO increase indicated by the DAF-2-DA fluorescence we used. Here the time factor is essential, whereas in the direct microscopic observation of NO-fluorescence release a continuous measurement from onset of PEMF till 15 minutes was possible; we look in flow cytometry at a time point of about 30 minutes after stimulation. On the other hand, in RT PCR we look nearly at the same time point after PEMF stimulation. Regarding mRNA expression, this may be a little too early for the high amounts of eNOS in endothelial cells and possibly fitting to the relative smaller amount of iNOS; nevertheless, even in these both cases a small elevation was detectable after PEMF.

Still unknown are the mechanisms underlying the direct coupling of the electric field to the cells (see also [19]). On the other hand, the molecular mechanisms underlying the activation of eNOS are reviewed recently by Fleming [20].

Finally, Tepper et al. [21] showed a positive effect of PEMF on angiogenesis by enhanced production of fibroblast growth factor beta-2. This is also an important aspect for therapy, since angiogenesis is a process critical for successful healing in various tissues.

For clinical applications (based upon multicenter, randomized, and prospective clinical studies) the Federal Drug Administration, USA, approved pulsed PEMF as safe and effective for treating nonunions and for osteoporosis therapy [19].

Interestingly, exposure to a static magnetic field, as carried out with the MagCell device in the off-mode, failed to stimulate blood flow in a statistically significant way. This again supports the view that only alternating magnetic fields with their inherent capability to induce electric fields and currents are physiologically effective. In addition, this result questions permanent magnets as an effective tool for biomedical stimulation.

## Figures and Tables

**Table:** (a)

Number of volunteers	14
Female	8
Male	6
Range of age (years)	25–55

**Table:** (b) Data identification

A	Control (device removed)
B	During treatment with a static magnetic field (device shutoff)
C	During treatment with an alternating magnetic field (device in operation)
D	Immediately after treatment (C)
E	Two minutes after treatment (C)

**Table:** (c)

Microcirculation in % of control (100%)	A	B	C	D	E
Mean value	100	113.7	137.3	128.9	134.1
SD (standard deviation)		18.2	23.6	20.3	26.6
SEM (standard error of the mean)	0.0	5.5	7.1	5.4	8.0

**Table:** (d) Statistical analysis (Student-Newman-Keuls Multiple Comparisons Test)

Comparison	Mean diff.	*q*	*P* value	Level of significance
A versus C	−37.27	6.68	*P* < 0.001	∗ ∗ ∗
A versus E	−34.09	6.11	*P* < 0.001	∗ ∗ ∗
A versus D	−28.85	5.40	*P* < 0.01	∗ ∗
A versus B	−13.73	2.46	*P* > 0.05	ns
B versus C	−23.55	4.06	*P* < 0.05	∗
B versus E	−20.36	3.51	*P* < 0.05	∗
B versus D	−15.12	2.77	*P* > 0.05	ns
D versus C	−8.43	1.55	*P* > 0.05	ns
D versus E	−5.25	—	*P* > 0.05	ns
E versus C	−3.18	—	*P* > 0.05	ns

**Table 2:** Mean values of all measurements (*n* = 9 cultures in controls and measurements, each with 5–9 ROIs, means 56 points of measurements).

		0 min	15 min	In %
PEMF	Mean	178,63	219,99	1,24
	SD	43,82	58,10	0,16
	*n*	56	56	56

control	Mean	165,62	161,10	0,98
	SD	31,84	37,61	0,13
	*n*	57	57	57
